# A Potential ceRNA Network for Neurological Damage in Preterm Infants

**DOI:** 10.1155/2021/2628824

**Published:** 2021-08-21

**Authors:** Jin Huang, Xuejing Liang, Zhenyu Cai

**Affiliations:** Department of Obstetrics and Gynecology, Aviation General Hospital of China Medical University, Beijing, China

## Abstract

**Objective:**

This study is aimed at identifying key genes involved in neurological damage in preterm infants and at determining their potential circRNA-miRNA-mRNA regulatory mechanisms.

**Methods:**

Differentially expressed miRNAs, mRNAs, and circRNAs were downloaded from the GEO database. GO and KEGG enrichment analyses were used to determine possible relevant functions of differentially expressed mRNAs. The TTRUST database was used to predict differential TF-mRNA regulatory relationships. Then, CircMIR, miRDB, TargetScan and miRTarBase were then used to map circRNA/miRNA-TF/mRNA interaction networks. Finally, GSEA enrichment analysis was performed on the core transcription factors.

**Results:**

A total of 640 mRNAs, 139 circRNAs, and 206 differentially expressed miRNAs associated with neurological injury in preterm infants were obtained. Based on the findings of Cytoscape and PPI network analysis, the hsa_circ_0008439-hsa-mir-3665-STAT3-MMP3 regulatory axis was established. GSEA analysis revealed that suppressed expression levels of STAT3 were associated with upregulated oxidative phosphorylation pathways in the neurological injury group of preterm infants.

**Conclusions:**

The circRNA-miRNA-TF-mRNA regulatory network of neurological injury in preterm infants can be used to elucidate on the pathogenesis of brain injury and help us with the early detection of brain injury in preterm infants.

## 1. Introduction

Infants born at less than 37 weeks of gestational age are referred to as premature and often have a birth weight of less than 2500 g. Due to the immaturity of their systemic organs, they are far more likely to suffer from complications when compared to full-term babies, especially neurological damage [1]. About 10% of preterm infants may have varying degrees of motor impairments [2] while about 25-50% present with cognitive impairments [3], visual and auditory impairments [4], and social behaviour, attention, and learning deficits [5, 6]. With advances in medical technology, the survival rates of preterm babies have significantly increased; however, incidences of neurological complications have not exhibited a proportional decrease [7]. Moreover, mechanisms involved in the development of neurological damage in preterm infants have not been elucidated. Studies have evaluated the roles of inflammatory factors [8], oxidative stress [9], and the amino acid accumulation [10], in the molecular mechanisms of neurological damage; however, findings have not been conclusive. Therefore, elucidation of the molecular mechanisms involved in neurological damage in preterm infants will inform the development of appropriate diagnostic and therapeutic options.

miRNAs, as noncoding RNAs, act by targeting proteins [11] and are involved in disease development through the ceRNA mechanism [12]. Sequenced peripheral blood of pregnant mothers of preterm infants was found to exhibit differences in expressions of 164 miRNAs, compared to full-term infants [13]. miRNAs regulate protein expression levels mainly by targeting with mRNAs. Analysis of placental tissues from preterm infants revealed a number of mRNAs that are associated with brain damage [14]. Another similar study sequenced fetal placental tissue, grouped according to the presence or absence of cognitive impairment, found 117 differentially expressed mRNAs and identified significantly affected pathways including oxidative phosphorylation, Parkinson's disease, and Alzheimer's disease [15]. In addition to its targeted regulatory relationship with mRNA, miRNA can also form ceRNA, circRNA-miRNA-mRNA, or a competitive binding miRNA mechanism of circRNA-miRNA-lncRNA to mRNA and genes including circRNA and lncRNA, which is thought to play an important role in biological behaviour. Comparisons of peripheral blood circRNA sequencing data from patients with premature ventricular white matter injury revealed 119 differentially expressed circRNAs, with the same preliminary construction of a miRNA-circRNA network [16]. The above studies revealed differential genetic changes during brain injury among preterm infants, with the exact mechanisms being unclear.

Therefore, we downloaded three datasets from the GEO database, including one mRNA dataset (GSE61822), one miRNA dataset (GSE106224), and one circRNA dataset (GSE131475). Using the downloaded datasets, we used bioinformatics approaches to explore the probable molecular mechanisms of neurological damage in preterm infants.

## 2. Methods

### 2.1. Data Download and Preprocessing

Gene expression transcriptomic microarray data for chorioamniotic membrane samples of placental tissue in preterm infants with and without neurological impairment, along with the corresponding clinically relevant information data, were downloaded from GSE61822 [15]. Peripheral blood miRNA expression data for pregnant women who had and for those who did not have preterm labour were obtained from the GSE106224 dataset [13]. The miRNA expression matrix of peripheral blood between the two groups and the corresponding clinical information was downloaded collated from the GSE106224 dataset. The GSE131475 dataset was used to compare circRNA expression levels in peripheral blood of preterm infants with periventricular white matter damage (PWMD) and without damage. [16].

### 2.2. Analysis of Variances

Based on the microarray platform file, the mRNA microarray gene IDs were converted to gene symbol and further logarithmically transformed to obtain the mRNA gene expression matrix. Based on paired information from clinical samples, a moderated paired *t*-test was performed on the mRNA expression matrix using the limma package based on paired information from clinical samples [17]. The thresholds, *p* < 0.05 and ∣log_2_FC | >0.3(FC > 1.35), were used to filter differentially expressed mRNAs between the two groups. To obtain differentially expressed miRNAs and a corrected miRNA expression matrix, differential analysis of miRNA expression matrix was performed using the edgeR package [18]. For the circRNA expression matrix of GSE131475 dataset, samples with more than 80% missing values were excluded, and circRNAs with missing values were deleted. The collated expression matrix was log-transformed after which the limma package was used to analyze the differences between the two groups based on the paired information of the clinical samples. Filtering of differentially expressed miRNAs and circRNAs expression between the two groups was performed at the threshold of  *p* < 0.05 and FC > 1.5.

Heat mapping of differentially expressed mRNAs, miRNAs, and circRNAs was, respectively, performed using pheatmap and gplots packages. Plot function in the base package was applied to make a volcano plot on the above variance expressions.

### 2.3. Enrichment Analysis

Based on up- and downregulated differentially expressed mRNA transcripts, functional GO and KEGG pathways enrichment analyses were performed for up- and downregulated genes, respectively. Enrichment analysis and drawing of bar graphs of GO and KEGG pathways were performed using the org.Hs.eg.db package and clusterProfiler [19]. The pathways were filtered at *p* < 0.05.

### 2.4. TF-mRNA Network Construction

The TF-mRNA relationship pair data were downloaded from the TTRUST database. Findings of the RNA differential analysis were intersected with the TF and corresponding mRNAs in the relationship pairs to obtain differential TF-mRNA regulatory relationship pairs. Mulberry mapping of differential TF-mRNA relationships was performed using the ggalluvial and ggplot2 packages.

### 2.5. Construction of miRNA-TF-mRNA Regulatory Network

The miRNA-mRNA regulatory relationship pairs were downloaded from the miRDB database (http://mirdb.org/), TargetScan database (http://www.targetscan.org/vert_72/), and miRTarBase (https://mirtarbase.cuhk.edu.cn/) (php/index.php). The intersection was used to select the differentially expressed miRNAs and the corresponding target mRNAs that were present in the miRNA differential analysis results of the relationship pairs. Then, target mRNAs of differentially expressed miRNAs were intersected with the TF and mRNAs of the TF-mRNAs that had been constructed in the previous step. Then, the final miRNA-TF-mRNA regulatory relationship network was obtained.

### 2.6. Prediction of circRNA and miRNA Relationships

The miRNAs in the miRNA-TF-mRNA network obtained in the previous step and the differentially expressed circRNAs obtained from the differential analysis were predicted one by one using the CircMIR (http://www.bioinf.com.cn/?page_id=10) software. Using miRanda (http://www.microrna.org/microrna/getDownloads.do) and RNAhybrid (https://bibiserv.cebitec.uni-bielefeld.de/rnahybrid/) algorithms, the corresponding predictions were calculated for each pair of action relations, and the results are presented as circle plots.

### 2.7. Construction of circRNA-miRNA-TF-mRNA Interaction Network

The Cytoscape (version 3.8.2) software was used to map the circRNA-miRNA-TF-mRNA regulatory network. The MCODE plugin (degree cutoff = 2, node score cutoff = 0.2, k − core = 2, and max.depth = 100) was also applied in the establishment of core genes and subnetworks in the network. Based on their enrichment scores, the top two core subnetworks were selected for presentation and subsequent analysis based on their enrichment scores.

### 2.8. GSEA Gene Set Functional Enrichment Analysis

Based on the core subnetwork obtained, the TF factors within it were selected and used to construct the circRNA-miRNA-TF-mRNA axis that may be important in the regulation of neurological impairment. The selected core TF was analyzed using the GSEA software (version 4.1.0). Then, the selected gene sets “c2.cp.kegg.v7.4.symbols.gmt” were used for pathway enrichment annotation.

## 3. Results

**Figure 1:**
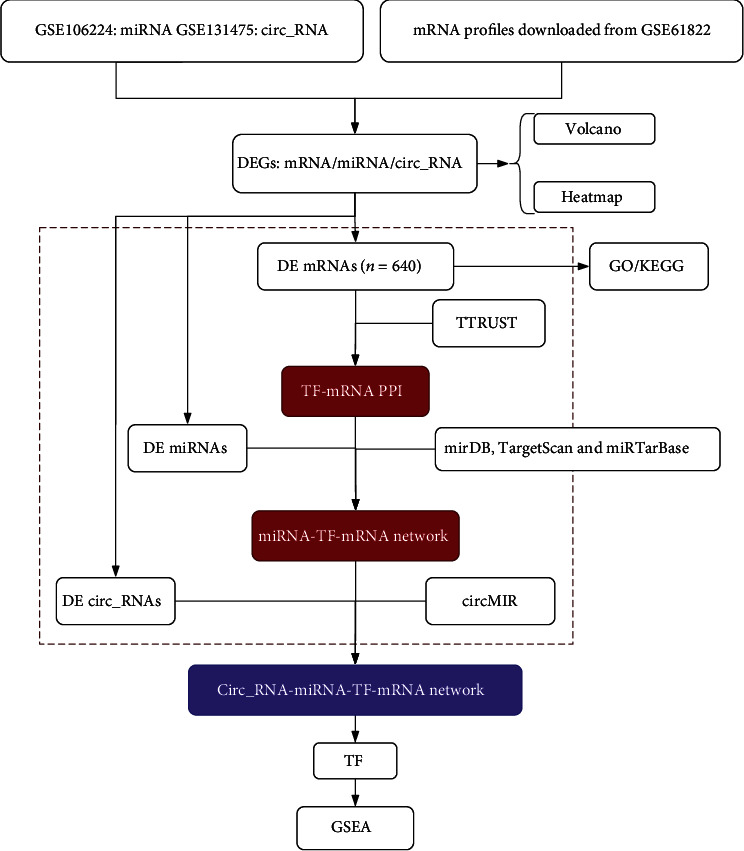
Schematic presentation of the analysis process.

A schematic presentation of this study is shown in Figure 1. The three datasets were used for the differential analysis of mRNA, miRNA, and circRNA, while the circRNA-miRNA-TF-mRNA network was constructed based on the results of database interactions.

### 3.1. Data Download

A total of 28 placental tissue transcriptomic data and the corresponding clinical information for preterm infants were downloaded from the GSE61822 dataset for preterm infants. Fourteen of these cases were associated with neurological impairments while and the other 14 were not. Peripheral blood miRNA expression matrices for 20 preterm and 50 nonpreterm pregnant women were downloaded from the GSE106224 dataset for miRNA analysis. circRNA expression data files for six preterm infants were downloaded from the GSE131475 dataset. Three of them had PWMD while the other three did not.

### 3.2. Variance Analysis

**Figure 2:**
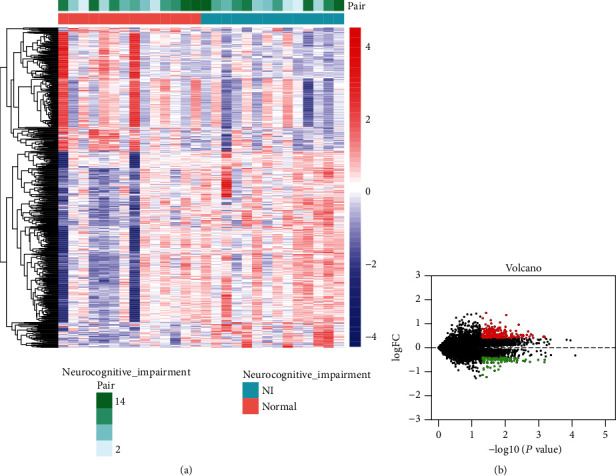
mRNA differential analysis and volcano plot results. (a) Heat map showing differential mRNA expression levels between the neurological impairment group and the normal group. (b) Volcano plot showing fold differences in gene expression between the two groups and *p* value relationship for the significance test. Red represents upregulated expression, and blue/green represents downregulated gene expression.

**Figure 3:**
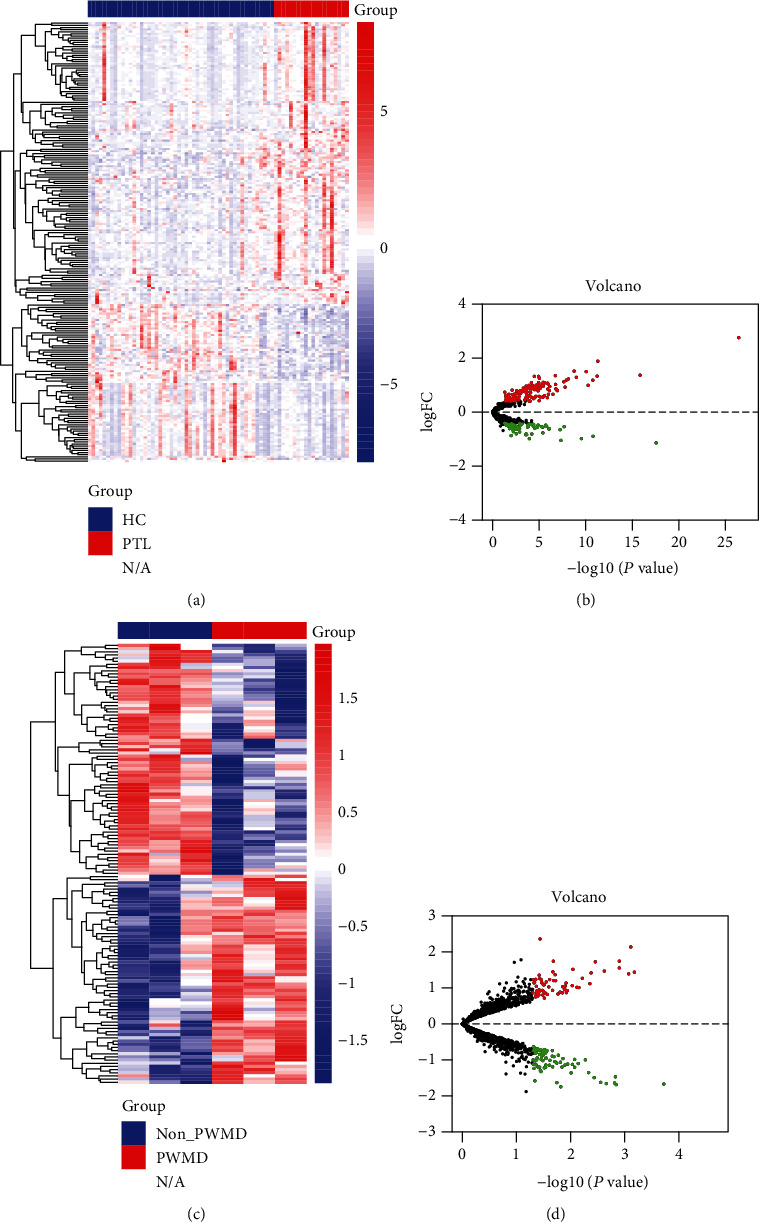
Results of differential analysis and volcano plot for miRNA and circRNA. (a) Heat map showing differences in miRNA expression levels in the peripheral blood of mothers in the normal delivery group and in the preterm group. (b). Volcano plot showing fold differences in miRNA expression levels between the two groups and the *p* value. (c) Heat map showing differences in circRNA expression in the peripheral blood of mothers in the two groups, with and without periventricular white matter injury. (d) Volcano plot showing fold differences in circRNA expression levels between the two groups.

Results of mRNA differential expression analysis are shown in Figure 2. A total of 640 differentially expressed mRNAs were obtained by filtering at *p* < 0.05 and FoldChange > 1.35. Results of miRNA and circRNA differential analysis are shown in heat and volcano plots (Figure 3), filtered by *p* < 0.05and FoldChange > 1.5 to obtain 206 DE miRNAs and 139 DE circRNAs, respectively.

### 3.3. Functional Enrichment Analysis

**Figure 4:**
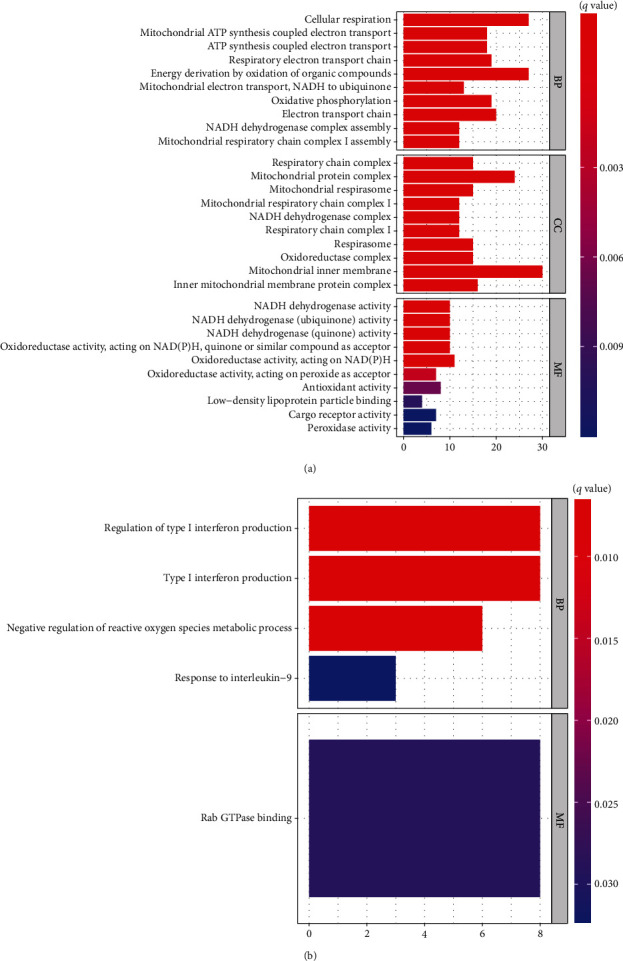
GO enrichment analysis of differential mRNA in preterm infants with and without neurological impairment. (a) GO enrichment pathways that were upregulated in placental tissues in the presence of neurological impairment. (b) GO enrichment pathways that were downregulated in placental tissues in the presence of neurological impairment. (c) GO enrichment pathways that were upregulated in placental tissue in the presence of neurological impairment.

**Figure 5:**
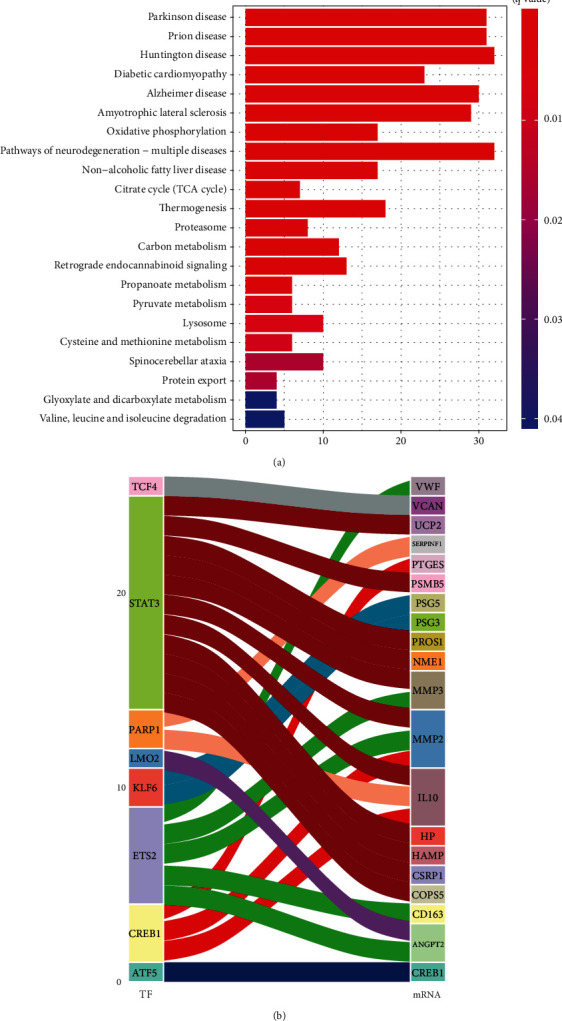
(a) KEGG enrichment analysis of differential mRNA in preterm infants with and without neurological impairment. KEGG enrichment pathways were found to be upregulated in the neurological impairment group. No downregulated enrichment pathways were found. (b) A Sankey diagram showing the predicted differential TF-mRNA regulatory relationships according to the TTRUST database.

The GO and KEGG pathways enrichment analyses for differentially expressed mRNA genes were performed.  Figure 4 shows GO pathways for up- and downregulated genes. In preterm infants with neurological impairments, five GO pathways with the most significant differences in upregulation were “cellular respiration,” “mitochondrial ATP synthesis coupled electron transport,” “ATP synthesis coupled electron transport,” “respiratory electron transport chain,” and “energy derivation by oxidation of organic compounds.” The five GO pathways with most significant differences in downregulation were “regulation of type I interferon production,” “type I interferon production,” “negative regulation of reactive oxygen species metabolic process,” “response to interleukin-9,” and “Rab GTPase binding.” KEGG pathway analysis (Figure 5(a)) revealed that the five most significant upregulated pathways were the “Parkinson disease,” “Prion disease,” “Huntington disease,” “diabetic cardiomyopathy,” and “Alzheimer disease”.

### 3.4. TF-mRNA Action Relationship

**Figure 6:**
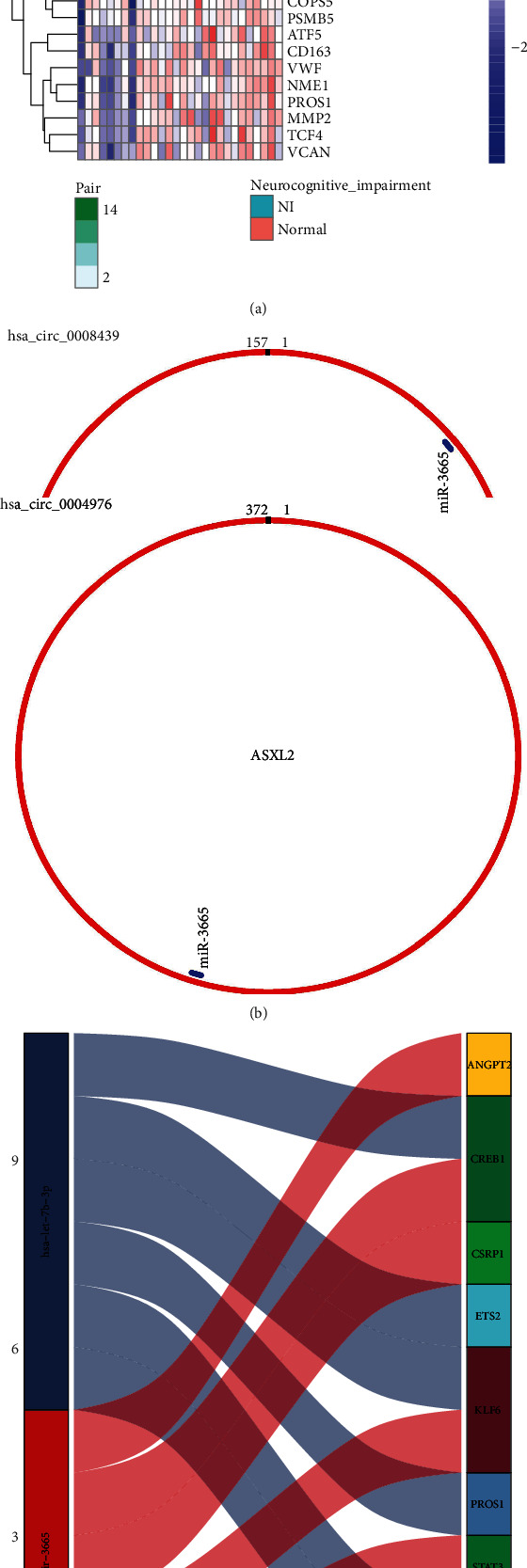
Construction of circRNA-miRNA-TF-mRNA regulatory network. (a) Heat map showing expression of individual gene transcripts in the TF-mRNA regulatory network with and without neurological impairment groups with differences. (b) Predicted results of differential miRNA as well as circRNA regulatory relationships according to CircMIR. (c) Results of miRNA and TF and mRNA (containing TF) regulation.

From the TTRUST database, a total of 27 TF-mRNA regulatory pairs were obtained after intersecting with differentially expressed mRNAs.  Figure 5(b) shows the relationship between mRNA expression levels of 8 TFs and their target genes.  Figure 6(a) shows the expression of individual gene transcripts in the TF-mRNA regulatory network with and without neurological impairments.

### 3.5. miRNA-TF-mRNA Action Relationship

The miRNA-mRNA regulatory relationships were extracted from the miRDB, TargetScan, and miRTarBase databases. From intersections in the above differential TF-mRNA regulatory network, 2 differential miRNAs and their corresponding target mRNAs (containing TF) were obtained.  Figure 6(c) shows the differential miRNA-mRNA/TF regulatory relationship.

### 3.6. circRNA-miRNA Relationships

The CircMIR software and its miRanda and RNAhybrid algorithms were used to predict miRNAs and differentially expressed circRNAs in the above miRNA-mRNA/TF regulatory relationship. Two circRNAs and 1 miRNA were obtained (Figure 6(b)). The corresponding analysis of the forecast results was reported in sup.tab1.

### 3.7. Network Construction and Subnetwork Construction

**Figure 7:**
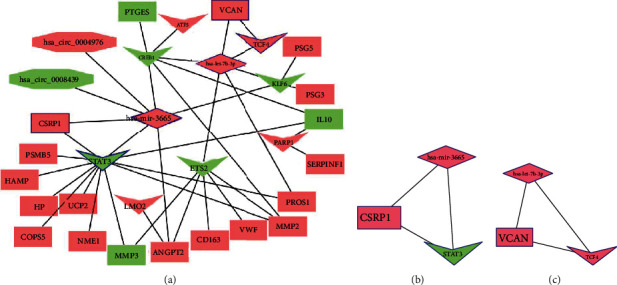
Protein interaction networks. (a) circRNA-miRNA-TF-mRNA regulated protein interaction networks. (b, c) Top 2 aggregator network modules predicted from MCODE. Rectangles represent mRNA, inner quadrilateral represents TF, rhombus represents miRNA, and octagon represents circRNA.

The Cytoscape software was used to map and construct the regulatory network for the above regulatory pairs (Figure 7(a)). Further analyses based on MCODE and filtering conditions resulted in 2 subnetworks and core genes (Figures 7(b) and 7(c)).

### 3.8. GSEA Enrichment Analysis of Core Genes

**Figure 8:**
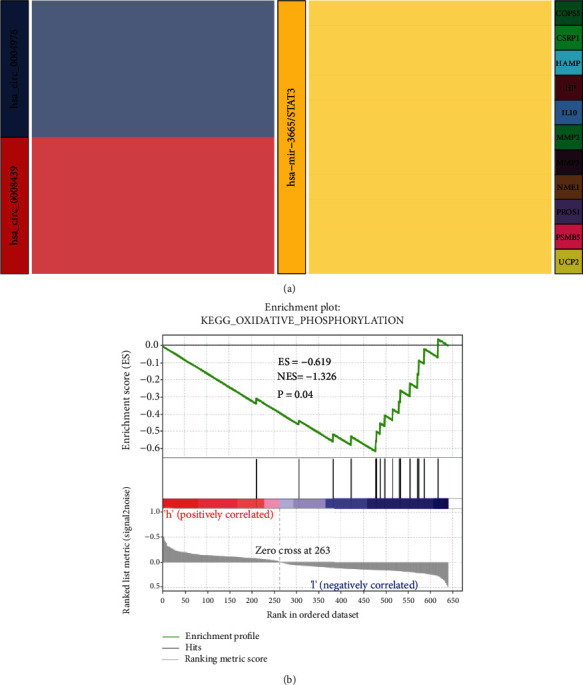
Potential hsa_circ_0008439-hsa-mir-3665-STAT3-MMP3 regulatory pathway and enrichment analysis. (a) A Sankey diagram showing the transcription factor, STAT3, in the core genes selected according to the subnetwork, demonstrating the circRNA-STAT3 regulatory network constructed around hsa-mir-3665/STAT3. (b) Results of GSEA enrichment analysis of selected STAT3 transcription factors showing enrichment in the lower STAT3 expression group upon KEGG_OXIDATIVE_PHOSPHORYLATION (*p* = 0.04).

Based on the obtained transcription factor STAT3 in the aggregated subnetwork and the corresponding ceRNA regulatory rules, we constructed the hsa_circ_0008439/hsa-mir-3665/STAT3/MMP3 regulatory axis (hsa_circ_0008439 and MMP3 were selected because, together with STAT3, they were downregulated in the network and, according to ceRNA rules, are more likely to be within the same regulatory axis) (supplementary table 1). A related regulatory axis was further constructed around hsa-mir-3665/STAT3 (Figure 8(a)). GSEA analysis was performed on high- and low-expressed STAT3.  Figure 8(b) shows that STAT3 expression was suppressed in the group of preterm infants with neurological impairments, while the KEGG_OXIDATIVE_PHOSPHORYLATION pathway was upregulated (*p* = 0.04). This pathway may have a role in regulation of neurofunctional development in preterm infants.

## 4. Discussion

Due to advances in perinatal medicine, survival outcomes for preterm babies have considerably improved, especially for very early preterm babies. However, not only does late complications of brain injury in preterm babies cause suffering to the child, they also place a great burden on the family and society [20]. Therefore, it is extremely important to establish the molecular mechanisms of brain damage in preterm infants. In addition, most studies nowadays are evaluating the miRNA-mRNA and TF-mRNA regulatory networks, and there are fewer studies on regulatory networks of TF and miRNA. We used the differentially expressed mRNA; miRNA constructs the miRNA-TF/mRNA regulatory network, which we combined with circRNA to synthesize the circRNA-miRNA-TF-mRNA regulatory network. These networks may be helpful in disease diagnosis and treatment.

We identified a total of 640 mRNAs that were differentially expressed in preterm infants with brain injury. The GO and KEGG pathway enrichment analyses of differentially expressed mRNA genes revealed that the five GO pathways that were most significantly differentially upregulated in preterm infants with neurological impairments were “cellular respiration,” “mitochondrial ATP synthesis coupled electron transport,” “ATP synthesis coupled electron transport,” “respiratory electron transport chain,” and “energy derivation by oxidation of organic compounds.” Neural damage in preterm infants were found to be associated with energy metabolism. It has been suggested that the function of the mitochondria is related to that of the hippocampus [21]. Moreover, reducing energy metabolism suppresses white matter damage in the brain [22]. Five downregulated significant GO pathways were also revealed, including “regulation of type I interferon production,” “type I interferon production,” “negative regulation of reactive oxygen species metabolic process,” “response to interleukin-9,” and “Rab GTPase binding.” These findings show that inflammatory pathways are involved in neurological damage in preterm infants. The KEGG pathway analysis revealed that the five most significantly upregulated pathways were the “Parkinson disease,” “Prion disease,” “Huntington disease,” “diabetic cardiomyopathy,” and “Alzheimer disease.” Early brain damage in preterm infants is not usually characterized by specific features [23] and is not detectable. The later stages of neurological damage are often more noteworthy. The above results reveal that molecules associated with brain damage in preterm infants are also involved in other neurological disorders; therefore, preterm birth is a risk factor for neurological damage in later life.

For differentially expressed mRNAs, we used the TTRUST database to obtain TF-mRNA, through a classical mode of action, in which we obtained 27 pairs of TF-mRNA regulatory relationship pairs. Combining differentially expressed miRNAs, circRNA, we obtained 2 differentially expressed miRNAs and 9 mRNAs/TFs. Based on one of the miRNAs, miR-3665, circ_0004976-miR-3665, and miR-3665-ASXL2 regulatory axes were achieved. One of the key molecules in the aforementioned regulatory axis, miR-3665, which is associated with neurocognitive function in addition to tumour development, is involved in the development of neurological functions [24]. miR-3665 targets the regulatory protein, ASXL2, which can link various histamine modifying enzymes and link transcription factors to specific genetic loci by encoding a series of epistatic regulators [25]. This gene plays an important role in neurological development, cardiac function, adipogenesis, and cell formation [26]. Therefore, miR-3665 may be an important miRNA molecule that is involved in the mechanism of neurological injury in preterm infants.

Combining the above differentially expressed mRNAs, miRNAs, and circRNAs, PPI network analysis was performed to construct two miRNA-TF-mRNA networks, has-miR-3665-STAT3-CSPR1 and has-let-7b-3p-TCF4-VCAN. It has been reported that let-7b is downregulated in children with autism [27] and that it can target STAT3 and inhibit hippocampal glial cell activation in epilepsy [28]. Regarding its target protein, TCF3 has been found to be involved in neurodevelopment, and it inhibits neuroectodermal differentiation in mice [29]. miR-3665 is closely associated with neurodevelopment, and its target is STAT3, an important transcription factor involved in many biological processes, including neurodevelopment [30]. Therefore, we targeted the transcription factor, STAT3, and combined it with ceRNA regulatory rules to establish the hsa_circ_0008439-hsa-mir-3665-STAT3-MMP3 regulatory axis. The final effector, MMP3, accelerates the inflammatory process to promote neurodevelopmental damage. In addition, through GSEA analysis, oxidative phosphorylation was found to be upregulated in low-expression STAT3. Oxidative phosphorylation is associated with neurological damage and is involved in the development of various neurological disorders [31–33]. From the above findings, we identified molecular mechanisms regulating the hsa_circ_0008439-hsa-mir-3665-STAT3-MMP3 axis that may be associated with neurological damage in preterm infants. However, given the complexity of the regulatory network, further in vitro and in vivo experiments are needed to validate its possible regulatory mechanisms as a novel diagnostic marker. Experimental validation will elucidate on the mechanisms involved in neurological damage in preterm infants and provide a theoretical basis for disease diagnosis and treatment.

## 5. Conclusion

We identified 620 mRNAs, 206 miRNAs, and 139 circRNAs that are differentially expressed in preterm infants with neurological injury. Based on regulatory network analysis, two key has-miR-3665 and has-let-7b-3p were derived. Coregulatory relationship of hsa_circ_0008439-hsa-mir-3665-STAT3-MMP3 was derived in conjunction with the key transcription factor, STAT3. However, this study has some limitations. The small sample size of this study was subject to significant sampling error, and therefore, the sample size should be expanded in future studies to increase data credibility. Besides, this study is also limited by the lack of experimental validation of the regulatory relationships and functions of the above key genes. It is clear that the complexity of the regulatory mechanisms does not allow for direct clinical use of regulatory axes we have identified for diagnosis and treatment but requires more practical support from in vivo and ex vivo experiments.

## Data Availability

Raw data and codes are available on request from the corresponding author.

## Supplementary Materials

Supplementary Materials Table S1: hsa-miR-3665 vs. hsa_circ_0008439.
