# Supplementary material for: A Potential ceRNA Network for Neurological Damage in Preterm Infants
Source: Biomed Res Int. 2021 Aug 21;2021:2628824. doi: 10.1155/2021/2628824 (PMC8405308; doi:10.1155/2021/2628824)
Supplement: Supplementary Materials — Table S1: hsa-miR-3665 vs. hsa_circ_0008439. [file 2628824.f1.doc]

Supplementary table 1. hsa-miR-3665 vs hsa_circ_0008439

| **hsa-miR-3665 vs hsa_circ_0008439** |  |
| --- | --- |
| miRanda | RNAhybrid |
| Score: 140 | p-value: 0.03216 |
| Energy: -12.35 kCal/Mol | mfe: -23.5 kCal/Mol |
| Position: 22,39 | Position: 17,39 |
| miRNA 3' GCGGCGGGGCGUGGACGA 5' | miRNA 3' GCGGCGGGGC-------GUGGACGA 5' |
| ||||||| | :||:|: ||||||| |
| circRNA 5' CTATTAAGTTCACCTGCA 3' | circRNA 5' ATGCTCTATTAAGTTCACCTGCA 3' |
